# Corticomuscular and intermuscular coherence are correlated after stroke: a simplified motor control?

**DOI:** 10.1093/braincomms/fcad187

**Published:** 2023-06-17

**Authors:** Célia Delcamp, David Gasq, Camille Cormier, David Amarantini

**Affiliations:** ToNIC, Toulouse NeuroImaging Center, Université de Toulouse, Inserm, Université Paul Sabatier, 31062 Toulouse, France; ToNIC, Toulouse NeuroImaging Center, Université de Toulouse, Inserm, Université Paul Sabatier, 31062 Toulouse, France; Department of Functional Physiological Explorations, University Hospital of Toulouse, Hôpital de Rangueil, 31400 Toulouse, France; ToNIC, Toulouse NeuroImaging Center, Université de Toulouse, Inserm, Université Paul Sabatier, 31062 Toulouse, France; Department of Functional Physiological Explorations, University Hospital of Toulouse, Hôpital de Rangueil, 31400 Toulouse, France; ToNIC, Toulouse NeuroImaging Center, Université de Toulouse, Inserm, Université Paul Sabatier, 31062 Toulouse, France

**Keywords:** common central drive, motor control, neuromuscular network, stroke

## Abstract

During movement, corticomuscular coherence is a measure of central-peripheral communication, while intermuscular coherence is a measure of the amount of common central drive to the muscles. Although these two measures are modified in stroke subjects, no author has explored a correlation between them, neither in stroke subjects nor in healthy subjects. Twenty-four chronic stroke subjects and 22 healthy control subjects were included in this cohort study, and they performed 20 active elbow extension movements. The electroencephalographic and electromyographic activity of the elbow flexors and extensors were recorded. Corticomuscular and intermuscular coherence were calculated in the time–frequency domain for each limb of stroke and control subjects. Partial rank correlations were performed to study the link between these two variables. Our results showed a positive correlation between corticomuscular and intermuscular coherence only for stroke subjects, for their paretic and non-paretic limbs (*P* < 0.022; Rho > 0.50). These results suggest, beyond the cortical and spinal hypotheses to explain them, that stroke subjects present a form of simplification of motor control. When central-peripheral communication increases, it is less modulated and more common to the muscles involved in the active movement. This motor control simplification suggests a new way of understanding the plasticity of the neuromuscular system after stroke.

## Teaser

Delcamp *et al*. demonstrated for the first time a correlation between corticomuscular and intermuscular coherences in stroke patients, for their paretic and non-paretic limbs. This innovative result provides information on the modification of the neuromuscular system, which appears to be simplified for both hemi-bodies of stroke patients.

## Graphical Abstract

**Graphical Abstract:**
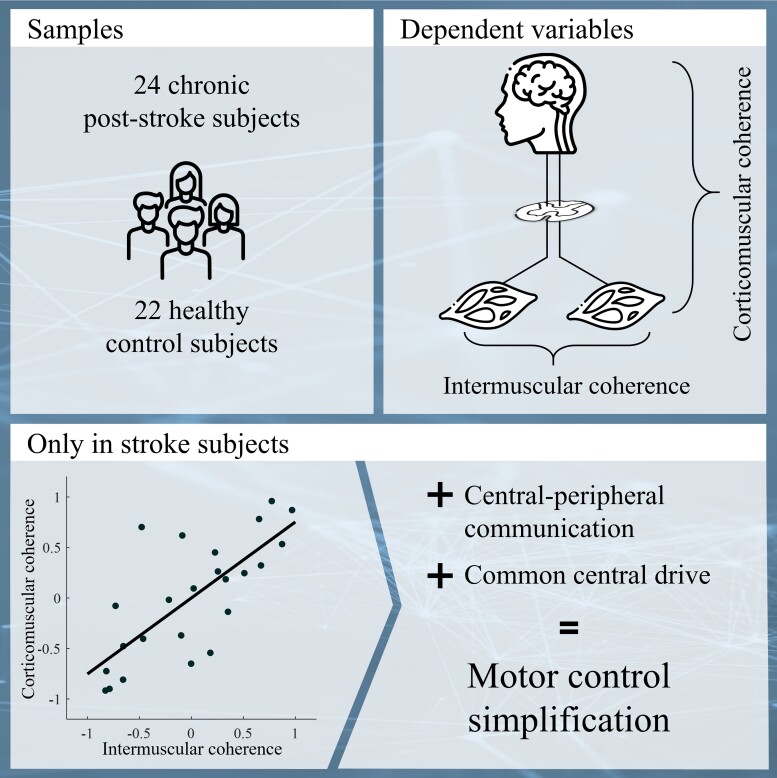

## Introduction

The study of motor control can be done in an ecological and non-invasive way by studying the communication between brain and muscles or between muscles.^1^ For this purpose, an oscillatory coupling is calculated between signals of cortical and muscular origin (EEG-EMG) to quantify corticomuscular coherence (CMC) or between two signals of muscular origin (EMG-EMG) to quantify intermuscular coherence (IMC). While CMC calculated in the beta (β ≈ 13–31 Hz) frequency band represents the bidirectional communication between brain and muscles,^2,3^ some studies calculating the phase delay between the EEG and the EMG signal, suggest that β-CMC largely reflects the involvement of central mechanisms in the control of voluntary motor activity through the corticospinal tract.^1,2^ IMC, for its part, makes it possible to assess the neuromuscular functional connectivity underlying the regulation of coordination between muscles.^4^ Although studies have also shown an effect of afferents on IMC during movement,^5,6^ the common drive theory suggests that the greater the β-IMC between two muscles, the greater the proportion of central control shared by these muscles.^7^ Thus, these two measures can provide information on the composition of the central neural drive.^8^

For stroke subjects, where motor control is altered in the chronic phase, CMC and IMC have previously been studied independently. β-CMC has been associated with impaired muscle synergies^9^ and with incomplete motor function.^10,11^ In addition to being associated with patients’ motor function, β-CMC also appears to be modified following stroke. Although there is no consensus on the direction of the difference in β-CMC between chronic stroke and healthy subjects, during movements, studies have either shown no difference in β-CMC between the two groups^12^ or an increase following stroke.^12-14^ Thus, the central descending drive, transmitted by the corticospinal tract could be impaired by stroke, reflected by a higher β-CMC. The motor function of stroke subjects has thus been correlated with the amount of β-CMC^11,15^ and when this tends towards that of healthy subjects, motor function may be improved.^14^ IMC alteration is also associated with impaired muscle synergies and incomplete motor function.^10,16^ Although few studies have investigated the effects of chronic stroke on β-IMC compared with healthy controls, for a agonist–antagonist muscle pair, β-IMC generally appears to be higher in stroke compared with healthy subjects^16-18^ as well as for a muscle pair consisting of two movement antagonist muscles.^16,17^ As with β-CMC, when β-IMC approaches physiological values, motor function improves in stroke subjects.^19^

In the literature, β-CMC and β-IMC may or may not be studied concomitantly. Although CMC and IMC do not appear to be directly associated in healthy subjects,^20,21^ these two parameters are often discussed together, since they both may reflect central descending drive.^14,22-24^ However, a possible link between these two variables has never been explored, although it would provide elements to better characterize the central descending drive of healthy and stroke subjects. Indeed, the link between a greater central descending drive (β-CMC) and a greater share of common central drive to synergistic muscles (β-IMC) is still unresolved, both in healthy and especially in stroke subjects during anisometric contractions. Answering this question would allow us to better understand the mechanisms of healthy and impaired motor control and the nature of the alteration of the central drive. Thus, in this study, we want to know whether or not central-peripheral communication is associated with the amount of common central drive and whether this possible relationship may be altered by stroke. Since both β-CMC and β-IMC are likely to represent central drive, it is possible that in healthy subjects, when the amount of central-peripheral communication increases, β-IMC also increases, possibly representing the share of common central drive sent to synergist muscles. In stroke subjects, there are concomitant changes in β-CMC and β-IMC in the same direction,^13,14,16,22,23^ we assume that a positive correlation can also exist between these two variables. Since the motor control of the muscles can vary according to their functional role,^1^ the study was done concomitantly for different muscle pairs.

## Materials and methods

### Participants

Twenty-four chronic stroke subjects [STROKE: 20 males/4 females; age: 57 years (33–76); Fugl–Meyer score: 40 ± 11; mean 3 years post stroke] and 22 healthy control subjects [CONTROL: 10 males/12 females; age: 50 years (20–73)] were included since 2018 (Table 1). Subjects were free of comprehension disorders, neurodegenerative diseases and pain in the upper limb during elbow extension movements. STROKE subjects were able to extend their elbow by at least 20° and were free of botulinum toxin in the elbow flexors for at least 4 months. Subjects were included in different protocols, which took place at the University Hospital of Toulouse, and which were conducted in accordance with the Declaration of Helsinki with the written informed consent of subjects at their entry into the protocol. Five stroke subjects and nine healthy control subjects were included in a study approved by the Research Ethics Committee of Toulouse University Hospitals (No. 07-0716). Eighteen stroke subjects were included in a routine care protocol (No. ID-RCB: 2017-A01616-47) and 1 stroke subject and 13 healthy control subjects were included in an interventional protocol (No. ID-RCB: 2018-000941-38).

**Table 1:** Detailed demographics and clinical of participants

Subjects	Sex	Age (years)	Time since stroke (months)	EmNSA/64	Upper limb Fugl Meyer/66	Stroke type	Stroke side, location
Controls (*n* = 22)	10 M/12F	50 (20–73)	/	/	/		
Stroke subjects	20 M/4F	57 (33–76)	38 ± 50	48 ± 14	40 ± 11		
1	M	61	51	27	38	Ischaemic	Right, cortical and subcortical territories of MCA
2	M	59	18	60	46	Haemorrhagic	Right, subcortical territories of MCA
3	F	69	19	50	44	Ischaemic	Right, Pons (paramedian)
4	M	65	75	50	32	Haemorrhagic	Left, basal ganglia and internal capsule
5	M	50	30	60	42	Ischaemic	Right, cortical and subcortical territories of MCA
6	M	57	14	61	45	Ischaemic	Left, posterior limb of the internal capsule
7	M	75	26	56	26	Ischaemic	Left, cortical and subcortical territories of MCA
8	M	48	8	33	49	Ischaemic	Left, cortical and subcortical territories of MCA
9	M	65	116	54	30	Ischaemic	Right, cortical and subcortical territories of MCA
10	M	49	13	62	53	Ischaemic	Predominant right, Pons (paramedian)
11	F	33	9	63	45	Ischaemic	Predominant left, Pons and middle cerebellar peduncles
12	F	34	12	48	21	Ischaemic	Left, subcortical territories of MCA
13	M	57	18	52	41	Ischaemic	Right, cortical and subcortical territories of MCA
14	M	56	34	26	29	Ischaemic	Right, cortical and subcortical territories of MCA
15	M	76	12	60	50	Ischaemic	Left, subcortical territories of MCA and hippocampus uncus
16	M	74	34	58	23	Ischaemic	Right, Pons (paramedian)
17	M	67	6	63	47	Ischaemic	Left, internal capsule
18	M	43	15	24	46	Ischaemic	Right, cortical and subcortical territories of MCA
19	M	72	43	39	36	Haemorrhagic	Right, thalamus
20	M	41	15	41	58	Ischaemic	Right, subcortical territories of MCA
21	M	52	36	64	41	Ischaemic	Left, internal capsule
22	M	54	12	23	27	Haemorrhagic	Right, internal capsule and thalamus
23	F	39	52	64	63	Ischaemic	Right, cortical and subcortical territories of MCA
24	M	54	27	35	47	Ischaemic	Right, cortical and subcortical territories of MCA

### Procedure

As previously described in details,^14^ participants were seated in front of a table with 80° shoulder flexion and 90° internal rotation. They performed 2 randomized sets of 10 self-paces active elbow extensions for each limb. Each elbow extension movement was followed by an elbow flexion to reach the initial position and each movement was separated by a rest period ranging from 8 to 15 s.

During movements, kinematic, electromyographic and electroencephalographic activity were recorded, respectively, at 125 Hz (system OptiTrack; NaturalPoint Inc., Corvallis, OR, USA), 1000 Hz (system OptiTrack; NaturalPoint Inc.) and 1024 Hz (64-electrodes cap; BioSemi instrumentation, Amsterdam, The Netherlands).^25^

The muscle activity recorded was that of the triceps brachii (TB) as the main elbow extensor and the biceps brachii (BB) and brachioradialis (BR) as the main elbow flexors.^26^ A verification procedure was performed to limit crosstalk among muscles.

### Data analysis

#### Pre-processing

Kinematic continuous data were low-pass filtered at 6 Hz. The detection of elbow extensions was done with a threshold of 0.01°/s on the angular velocity data. Electromyographic continuous data were 3–100 Hz band-pass and 49–51 Hz band-stop filtered.^11,14^ Electroencephalographic continuous data were 0.5–100 Hz band-pass and 49–51 Hz band-stop filtered^27^ and pre-processed with the Automagic toolbox ‘clean_rawdata’ pipeline (with the default setting).^28^ This procedure allows to:

- Reject the bad channel without variation in amplitude for at least 5 consecutive seconds and raw channels that have an amplitude at least 4 times greater than the mean standard deviation of the scalp and channels that do not correlate with the other channel.
- Interpolate the rejected channels using the eeg_interp function.
- Interpolate time windows with artefacts using the ‘artefact subspace reconstruction method’.
- Remove the independent components by subtracting them from the signal using the ‘multiple artefact rejection algorithm’.

Then, signal was re-referenced by a Laplacian filter to improve the signal-to-noise ratio.^29^

#### Data processing

**Figure 1:**
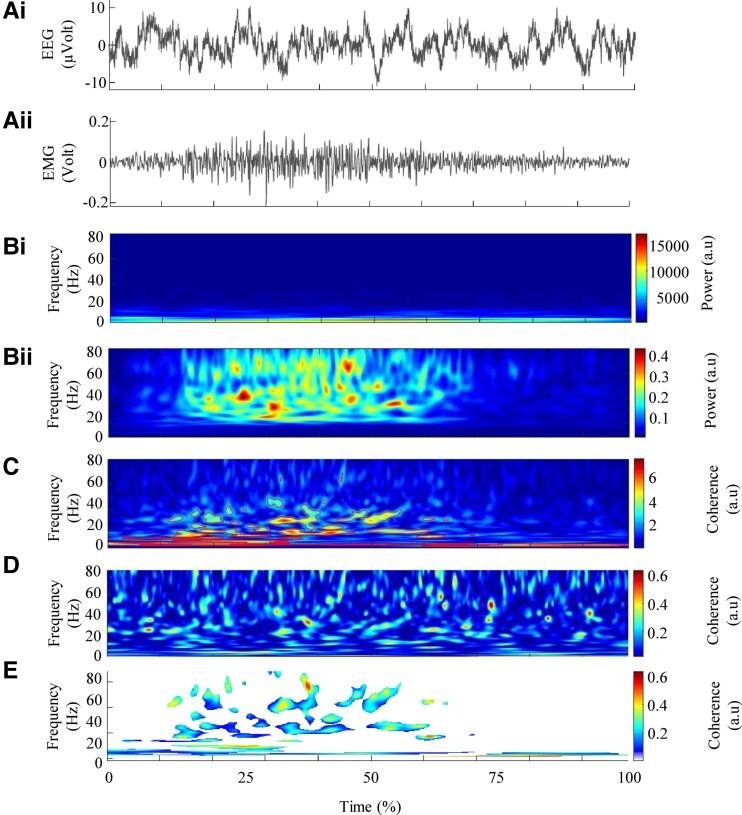
**Description of the different steps involved in the calculation of coherences, illustrated for the corticomuscular coherence.** Panel **Ai** represents the electroencephalographic activity of the C3 electrode (left) and Panel **Aii**, the electromyographic activity of the brachioradialis muscle (right) typical of a healthy subject (*n* = 1). Panels **Bi** and **Bii** represent the average auto-spectrum calculated from the respective electrophysiologic signals. Panel **C** represents the cross-spectrum and the red contours identify the areas in the time–frequency plane where the correlation between the electrophysiologic signals is significant. Panel **D** represents the wavelet magnitude-squared coherence between the two electrophysiologic time series. Panel **E** represents the wavelet magnitude-squared coherence where the correlation between the electrophysiologic signals is significant. For the calculation of intermuscular coherence, the processing steps are identical but with the input data in **A** being an electromyographic signal (for an illustration, see Charissou *et al*.,^32^ Figure 1).

For each subject, the continuous electromyographic and electroencephalographic data were segmented 3 s before and after each elbow extension, and CMC and IMC were calculated in time–frequency domain using Morlet wavelet approach introduced by Bigot *et al*.^30^ using *WavCrossSpec* software with the following steps:

1. After epoching each continuous electrophysiological signal of interest 3 s before and 3 s after each movement and normalized them to account for inter-movement time variability,^31^ the auto-spectra of each electroencephalographic and electromyographic signal were calculated. The parameters ‘nvoice’ (scale resolution of wavelets), ‘J1’ (number of scales) and ‘wavenumber’ (Morlet mother wavelet parameter) were, respectively, set to 7, 50 and 10 to yield accurate identification of oscillatory activity in the 0.23–79.97 Hz frequency range in 0.23 steps. These parameters set the time–frequency precision compromise to a 0.1 s–3 Hz precision window within the β (13–31 Hz) frequency band.
2. For each subject, the auto-spectrum obtained for the 20 movements was averaged to obtain an average auto-spectrum of the 20 movements for the EEG and EMG activity of each subjects (Fig. 1B).
3. The cross-spectrum between each interest signals was calculated (Fig. 1C).
4. CMC and IMC were calculated by normalizing the cross-spectrum by the product of the auto-spectrum as follows for an example of CMC calculation (Fig. 1D):
  $$R_{\mathrm{EMG}/\mathrm{EEG}}^{2}(\omega ,u)=\frac{{\left|S_{\mathrm{EMG}/\mathrm{EEG}}(\omega ,u)\right|}^{2}}{S_{\mathrm{EMG}}(\omega ,u)S_{\mathrm{EEG}}{\left(\omega ,u\right)}^{\prime }}$$
  where *S*_EMG/EEG_(*ω*, *u*) is the wavelet cross-spectrum between an EMG and an EEG time series at frequency *ω* and time *u*; *S*_EMG_(*ω*, *u*) and *S*_EEG_(*ω*, *u*) are wavelet auto-spectra of EMG and EEG time series at frequency *ω* and time *u*.
5. The significant coherence map was calculated using a binary mask of significance determined from the cross-spectrum and then applied to the coherence map to avoid retaining false positive coherence values (Fig. 1E).

To satisfy theoretical arguments regarding the calculation of coherence measures and to avoid subsequent inconsistencies in such results, the electromyographic signals have not been rectified.^30,33^

#### Corticomuscular coherence

The CMC analysis was carried out on the ipsilesional motor cortex (C3 or C4) and on the eight surrounding electrode clusters (e.g. C3: FC5, FC3, FC1, C5, C1, CP5, CP3, CP1). Then, in the ‘beta’ (β, 13–31 Hz) frequency band and in a time window of 200 ms before the velocity peak, the maximal CMC values among this nine-electrode cluster were computed for each time frame and frequency bin to calculate the volume under the significant area of this new coherence map.^30^ CMC data from the paretic limb of 11 post-stroke subjects and the non-dominant limb of 9 control subjects were already exploited in a previous study.^14^ However, in the current study, data from 14 post-stroke subjects and 13 control subjects were added. For all of these participants, data from the non-dominant limb of post-stroke subjects and dominant limb of control subjects were added.

#### Intermuscular coherence

The IMC was calculated for both the TB-BR (as a synergistic and agonist/antagonist muscle pair) and between the BB-BR (as synergistic and antagonist muscle pair) and quantified in the ‘beta’ frequency band and in the 200 ms window before the velocity peak as the volume under the significant areas.^30^ IMC data of post-stroke and control subjects included in this study were used in a previous study.^16^ However, the treatment of the non-dominant limb of control subjects was added to the current study.

The location of the time window was intended to limit the incorporation of feedback (e.g. information on body segment positions in space) into the quantification CMC,^34^ while the size of the window was optimized to reduce the variation in the elbow range of motion to reduce its potential effect on subject coherence.^35^ The variables were studied for the paretic and non-paretic limbs of STROKE and for the dominant and non-dominant limbs of CONTROL: IMC of the BB-BR muscle pair and the average of the β-CMC of the BB and the BR muscles as well as the IMC of the TB-BR muscle pair and the average of the β-CMC of the TB and BR muscles.

### Statistical analysis

Different variables can be directly related to β-CMC or β-IMC: age,^36,37^ motor function^10,11,14,15^ represented by antagonist co-contraction and coherence quantification parameters such as the average elbow angle and velocity during the quantification window.^16,17^

To consider these co-variables, partial correlations were performed between β-CMC and β-IMC for each muscle pair and limb of STROKE and CONTROL with the following co-variables: subjects’ age at protocol inclusion, level of antagonist muscle co-contraction (calculated by normalizing flexor muscle activity during elbow extension movements by their maximal activity during maximal voluntary contraction),^14^ the average elbow angle and velocity in the 200 ms quantification window of coherence. Because the residuals distribution of the model is non-normal (visual assessment and Shapiro–Wilk test), partial Spearman’s rank correlations were performed.

To compare the correlation between β-CMC and β-IMC of STROKE with that of CONTROL, a comparison of the Rho’s via Fisher transformations was performed between the paretic limb of STROKE and the dominant limb of CONTROL as well as between the non-paretic limb of STROKE and the dominant limb of CONTROL (for both muscle pairs).^38^

The alpha-risk threshold was set at 0.05. To control the false discovery rate, Benjamini–Hochberg procedure was performed and the critical *P*-values were calculated for each correlation and reported in Tables 2 and 3.^39^

**Table 2:** Partial correlations results: for stroke and control subjects for each of their two upper limbs and for the muscle pair biceps brachii–brachioradialis (BB-BR) and triceps brachii–brachioradialis (TB-BR)

Group	Limb	Muscle pair for IMC	Rho	95% CI	*P*-value	Benjamini–Hochberg critical *P*-value
Stroke subjects	Paretic	BB-BR	0.50	(0.10–0.78)	0.022*	0.025
		TB-BR	0.72	(0.36–0.91)	<0.001*	0.006
	Non-paretic	BB-BR	0.78	(0.48–0.91)	<0.001*	0.013
		TB-BR	0.59	(0.20–0.86)	0.005*	0.019
Control subjects	Non-dominant	BB-BR	0.35	(−0.21 to 0.68)	0.14	0.038
		TB-BR	0.57	(−0.27 to 0.90)	0.10	0.031
	Dominant	BB-BR	0.12	(−0.48 to 0.61)	0.63	0.044
		TB-BR	0.06	(−0.36 to 0.61)	0.82	0.05

The asterisks represent the tests that are significant regarding the Benjamini–Hochberg critical *P*-value.

**Table 3:** Correlation comparisons: between the paretic limb of STROKE and the dominant limb of CONTROL and between the non-paretic limb of STROKE and the dominant limb of CONTROL for the muscle pair biceps brachii–brachioradialis (BB-BR) and triceps brachii–brachioradialis (TB-BR)

Comparison	Muscle pair for IMC	Cohen *q*	*P*-value	Fisher *z*	Benjamini–Hochberg critical *P*-value
Paretic versus Dominant	BB-BR	0.429	0.088	1.354	0.05
	TB-BR	0.848	0.004*	2.677	0.025
Non-paretic versus Dominant	BB-BR	0.925	0.002*	2.921	0.012
	TB-BR	0.618	0.026*	1.95	0.375

The asterisks represent the tests that are significant regarding the Benjamini–Hochberg critical *P*-value.

## Results

**Figure 2:**
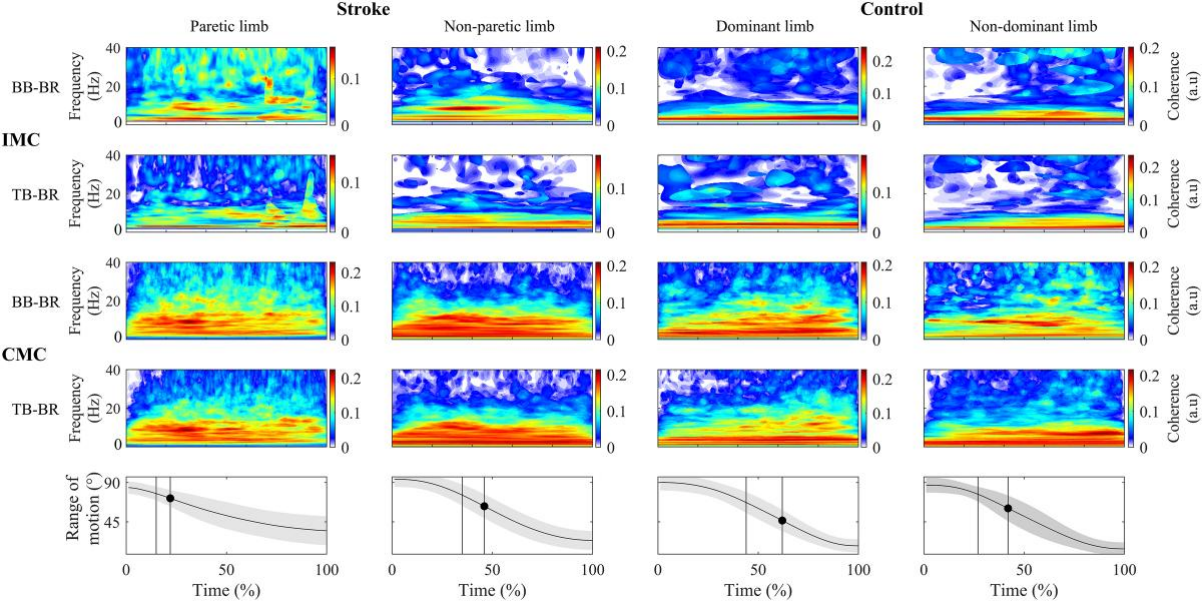
**Mean coherence maps of the paretic limb (first row) and non-paretic limb (second row) of stroke subjects (*n* = 24) and the dominant limb (third row) and non-dominant limb (fourth row) of control subjects (*n* = 22).** Intermuscular coherence (IMC) is represented for the biceps brachii–brachioradialis (BB-BR) (first line) and triceps brachii–brachioradialis (TB-BR) (second line) muscle pairs. The mean corticomuscular coherence (CMC) is represented between the biceps brachii and brachioradialis muscles (BB-BR) (third line) and triceps brachii and brachioradialis muscles (TB-BR) (fourth line). Please refer to Delcamp *et al*.^14,16^ for our previous investigation of the differences in CMC and IMC between stroke and control subjects. In the last line, the mean active range of motion during the movement is plotted with the standard deviation. The dot represents the velocity peak and the vertical lines the temporal quantification window.

For STROKE and CONTROL, β-CMC and β-IMC were significantly present (see Fig. 2 for data representation). For a detailed analysis of the differences in CMC and IMC between stroke and control subjects, please refer to Delcamp *et al*.^14,16^ Please note that an overview of (i) coherence power spectra and (ii) topographic representation of CMC for control and stroke subjects is provided in Supplementary Figs 1 and 2.

**Figure 3:**
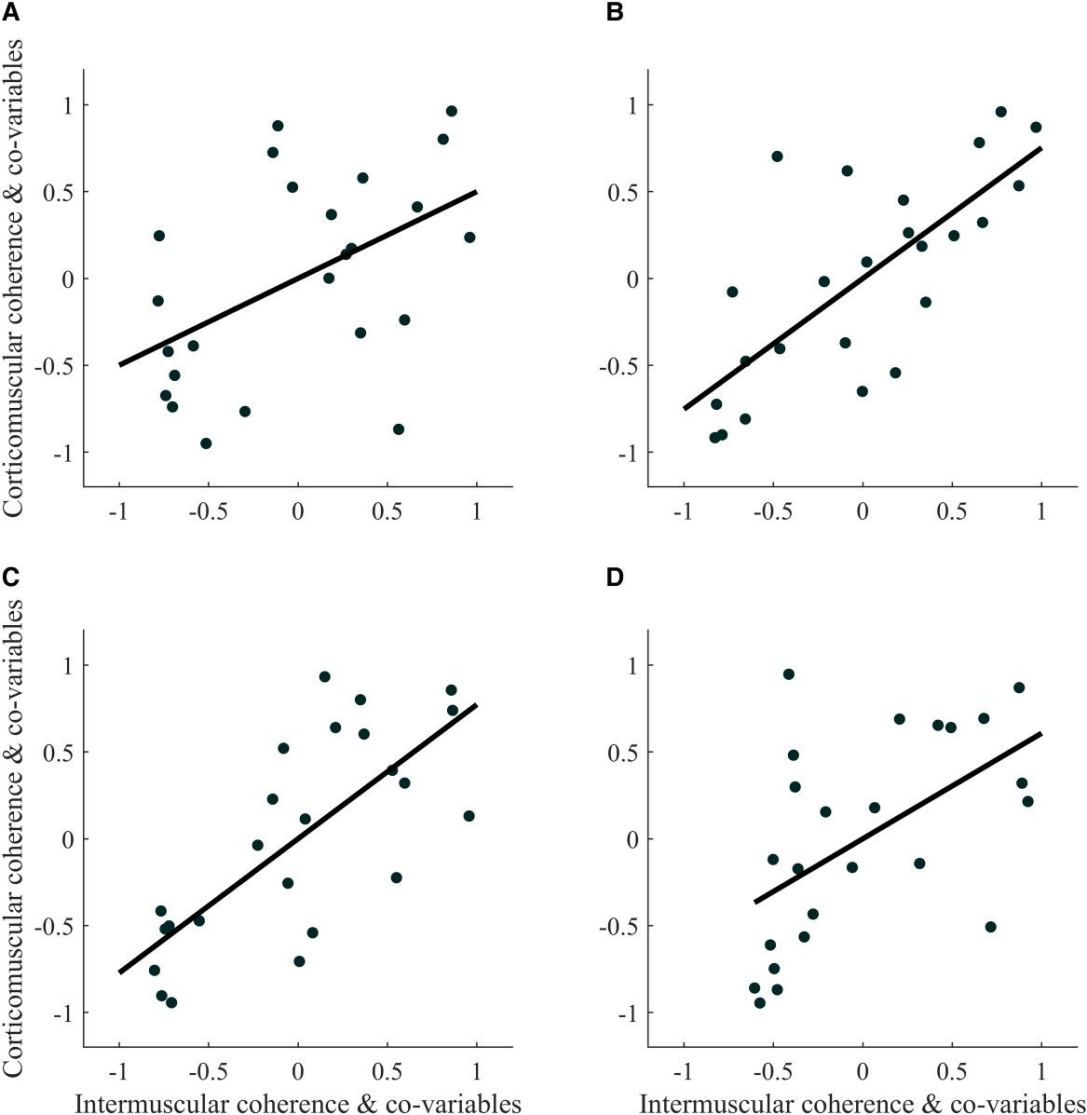
Partial rank Spearman’s correlation plot of (A) corticomuscular and intermuscular coherence for stroke subjects for the biceps brachii–brachioradialis [Rho = 0.50 (0.10–0.78); *P* = 0.022*] and (B) triceps brachii–brachioradialis muscle pairs [Rho = 0.72 (0.36–0.91); *P* ≤ 0.001*] of their paretic limb and (C) the biceps brachii–brachioradialis [Rho = 0.78 (0.48–0.91); *P* ≤ 0.001*] and (D) triceps brachii–brachioradialis muscle pairs [Rho = 0.59 (0.20–0.86); *P* = 0.005*] for their non-paretic limb. The data plotted are the residuals of the Spearman’s rank correlation performed between: intermuscular coherence and the co-variables, and corticomuscular coherence and the co-variables. The asterisks represent a significant correlation with respect to the Benjamini-Hochberg critical *P*-value.

**Figure 4:**
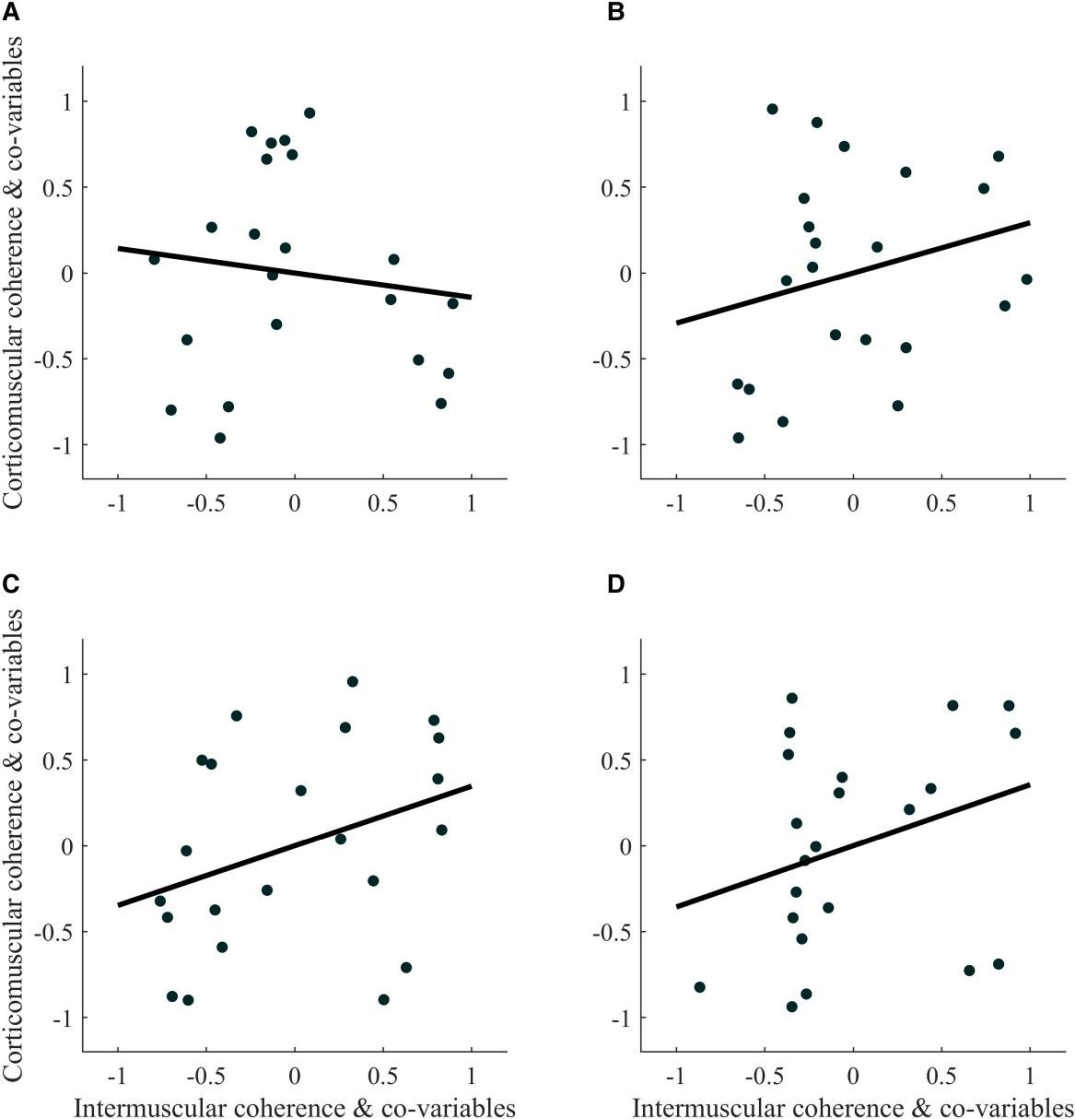
**Partial rank Spearman’s correlation plot of (A) corticomuscular and intermuscular coherence for control subjects for the biceps brachii–brachioradialis [Rho = 0.12 (−0.48 to 0.61); *P* = 0.63] and (B) triceps brachii–brachioradialis muscle pairs [Rho = 0.06 (−0.36 to 0.61); *P* = 0.82] of their dominant limb and (C) the biceps brachii–brachioradialis [Rho = 0.35 (−0.21 to 0.68); *P* = 0.14] and (D) triceps brachii–brachioradialis muscle pairs [Rho = 0.57 (−0.27 to 0.90); *P* = 0.10] for their non-dominant limb.** The data plotted are the residuals of the Spearman’s rank correlation performed between: intermuscular coherence and the co-variables, and corticomuscular coherence and the co-variables.

For both muscle pairs (BB-BR and TB-BR) of both limbs of STROKE (paretic and non-paretic), significant linear relationships were found between β-CMC and β-IMC [Rho > 0.50; 95% confidence interval (CI) = 0.10–0.78; *P* < 0.022; Fig. 3; Table 2], whereas no significant relationship was found between β-CMC and β-IMC in CONTROL (Rho < 0.57; 95% CI = −0.27 to 0.90; *P* > 0.10; Fig. 4; Table 2).

Comparison of the correlations between β-CMC and β-IMC revealed a significantly higher correlation for both muscle pairs of the non-paretic limb of STROKE compared with CONTROL (Fisher *z* > 1.95; Cohen *q* > 0.618; *P* < 0.026; Table 3). For the paretic limb of STROKE, the correlation for the TB-BR muscle pair is significantly higher than that of CONTROL (Fisher *z* = 2.677; Cohen *q* = 0.848; *P* = 0.004; Table 2), while that for the BB-BR muscle pair tends to be different (Fisher *z* = 1.354; Cohen *q* = 0.429; *P* = 0.088; Table 3).

## Discussion

This work reveals a significant relationship between β-CMC and β-IMC only for STROKE for both their paretic and non-paretic limbs and for agonist–antagonist and antagonist–antagonist muscle pairs. This correlation was significantly higher for both paretic and non-paretic limbs of STROKE compared with CONTROL. These results are consistent with a part of literature in stroke subjects where corticomuscular and IMC are modified in the same way for the paretic limb.^11,13,14,16^

In CONTROL, the absence of correlation between β-CMC and β-IMC allows to assume that the regulation of corticomuscular communication is less linked to the regulation of intermuscular communication during movements, neither for muscle pairs of agonist–antagonist muscles or antagonist–antagonist muscles nor for the dominant or the non-dominant limb. Thus, the intensity of central-peripheral communication is not directly related to the amount of common central drive directed to the muscles. These results are in agreement with previous work studying CMC and IMC during bimanual isometric contractions in healthy subjects^20,21^ which already suggested that IMC is not associated with CMC as it may represent something other than a central descending drive.

The correlations between β-CMC and β-IMC for the paretic limb of stroke patients show that stroke subjects with more corticomuscular communication also have more intermuscular communication. In other words, the greater the central-peripheral communication, the more it is common to the synergistic muscles. This simplification of motor control already mentioned in the literature^10^ appears then in the co-modulation of central-peripheral and intermuscular communication. This phenomenon may be explained by a cortical and a spinal hypothesis. Stroke alters the cortical activity of the injured hemisphere^40-42^ and thus the nature of the central descending drive (β-CMC)^11,14^ as well as the amount of common central drive sent to the synergist muscles (β-IMC).^16,18,23^ β-CMC and β-IMC could thus be co-modulated by this cortical alteration. Stroke also alters spinal inhibition mechanisms^43,44^ that may act as a filter on β-CMC and β-IMC.^45^ These filters altered by stroke would acts in the same way on the central descending drive and on the common central descending drive which could lead to co-modulated β-CMC and β-IMC. The main hypotheses presented above do not prevent us from making a complementary hypothesis according to which the increased afferents of the paretic limb could contribute to the correlation existing between β-CMC and β-IMC, since it is known that afferents can influence the β-CMC^2^ and β-IMC.^6,46^

Concerning the non-paretic limb of stroke patients, the observed correlation is in agreement with the literature where CMC and IMC are also altered in the non-paretic limb.^15,18,47^ In order to explain the correlation between β-CMC and β-IMC observed in the non-paretic limb, the same hypotheses as those outlined for the paretic limb can be evoked. Indeed, following stroke, the activity of the controlesional hemisphere is increased due to the decrease in interhemispheric inhibition of the injured hemisphere on the controlesional one.^48,49^ This increase in cortical activity could then lead to a similar modulation of the central descending drive as well as of the common central drive to the synergic muscles. Spinal inhibition mechanisms may also be altered in the non-paretic hemibody,^50^ which may act as a filter,^45^ also co-modulating the β-CMC and β-IMC.

## Conclusion

The above results on the co-modulation of β-CMC and β-IMC in stroke subjects when compared with control subjects allow us to discuss the impact of stroke on the simplified communication mechanisms to and between muscles of the paretic limb, which appear indistinguishable from the functional role of the muscles. In sum, regardless of pre-stroke handedness, which does not seem to influence coherence magniture,^51^ this work highlights a link between β-CMC and β-IMC for stroke patients, and suggests that the regulatory mechanisms of the neuromuscular communication network (where β-CMC and β-IMC are proportionally modulated) can be qualified as simplified. Given the relatively small sample size of this study, as well as the inclusion of stroke subjects with encephalic lesions of various locations (but altering the motor function of the upper limb), any generalization of these results must be made with caution. Anyway, these results suggest a new way of understanding the plasticity of the neuromuscular system of stroke subjects, with a simplification that seems to take place to allow movement despite the damage caused by stroke.

## Supplementary Material

fcad187_Supplementary_Data

## Data Availability

The data that support the findings of this study are the property of the University Hospital of Toulouse and may be available from D.G. upon reasonable request.

## Abbreviations

BB =: biceps brachii
BR =: brachioradialis
CMC =: corticomuscular coherence
IMC =: intermuscular coherence
TB =: triceps brachii
