# Supplementary material for: Corticomuscular and intermuscular coherence are correlated after stroke: a simplified motor control?
Source: Brain Commun. 2023 Jun 17;5(3):fcad187. doi: 10.1093/braincomms/fcad187 (PMC10292907; doi:10.1093/braincomms/fcad187)
Supplement: fcad187_Supplementary_Data [file fcad187_supplementary_data.pdf]

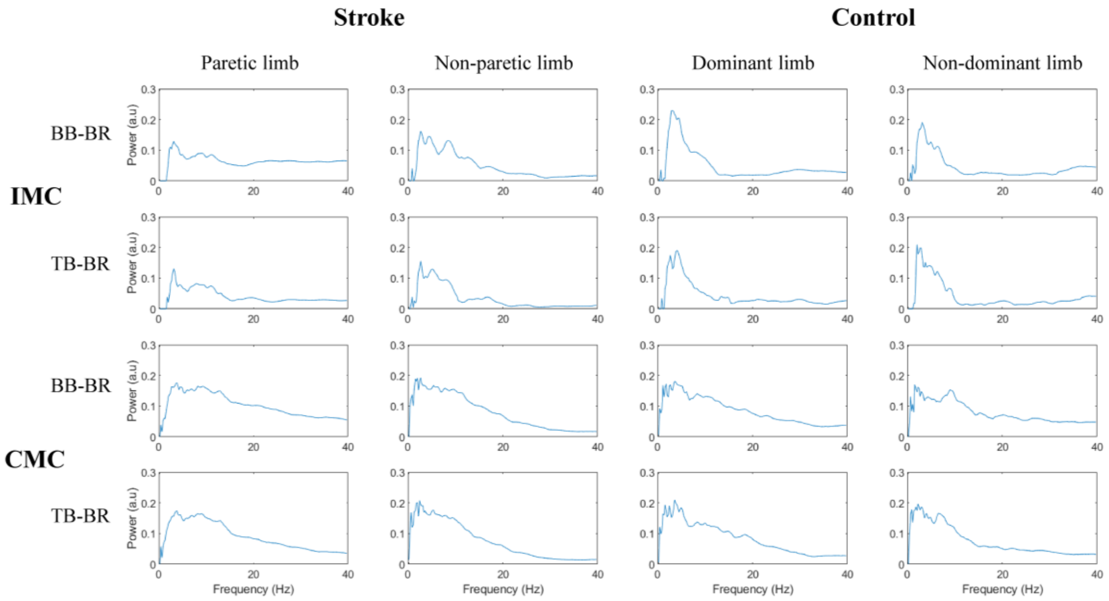

Supplementary Figure 1: Mean coherence power spectra for the paretic (**first row**) and the non-paretic (**second row**) limbs of stroke subjects (n=24), and the dominant (**third row**) and the non-dominant (**fourth row**) limbs of control subjects (n=22). Intermuscular coherence (IMC) is represented for the biceps brachii - brachioradialis (BB-BR) (**first line**) and triceps brachii - brachioradialis (TB-BR) (**second line**) muscle pairs. Corticomuscular coherence (CMC) is represented as the average of the CMC of the biceps brachii and brachioradialis muscles (BB-BR) (**third line**), and the average of the CMC of the triceps brachii and brachioradialis muscles (TB-BR) (**fourth line**).

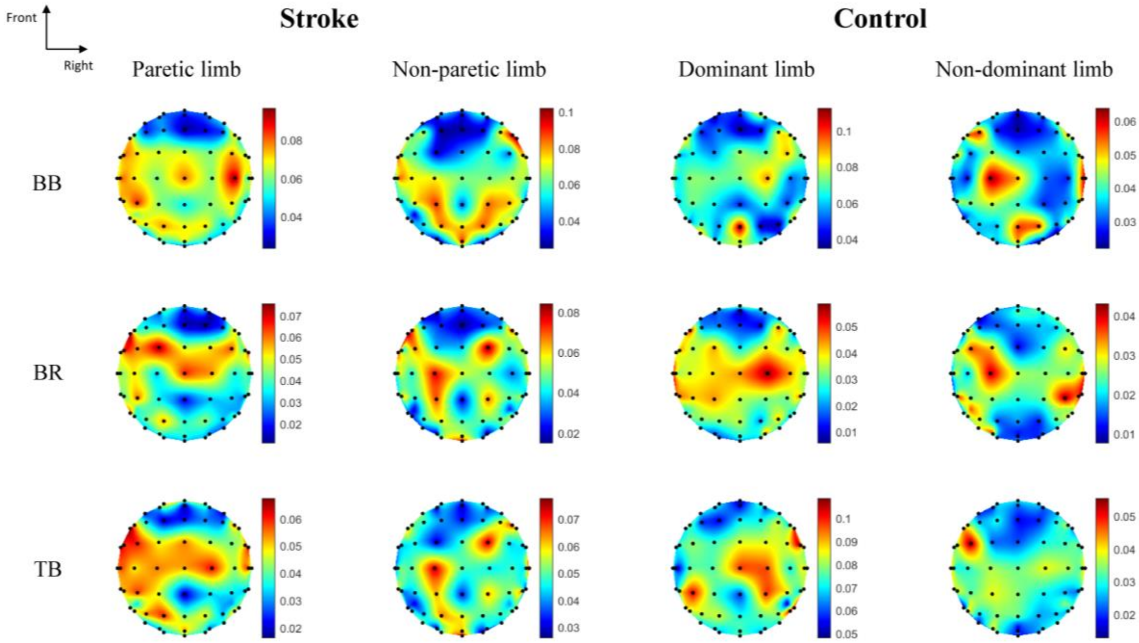

Supplementary Figure 2: Topographic representation of mean corticomuscular coherence in the beta band (β-CMC) for the paretic (**first row**) and the non-paretic (**second row**) limbs of stroke subjects (n=24), and the dominant (**third row**) and non-dominant (**fourth row**) limbs of control subjects (n=22). Corticomuscular coherence is represented for the biceps brachii (BB; **first line**), brachioradialis (BR; **second line**) and triceps brachii (TB; **third line**) muscles.
